# Unique spectrum of *SPAST *variants in Estonian HSP patients: presence of benign missense changes but lack of exonic rearrangements

**DOI:** 10.1186/1471-2377-10-17

**Published:** 2010-03-09

**Authors:** Mark Braschinsky, Riin Tamm, Christian Beetz, Elena Sachez-Ferrero, Elve Raukas, Siiri-Merike Lüüs, Katrin Gross-Paju, Catherine Boillot, Federico Canzian, Andres Metspalu, Sulev Haldre

**Affiliations:** 1 Department of Neurology, University of Tartu, Tartu, Estonia; 2 Institute of Molecular and Cell Biology, University of Tartu, Tartu, Estonia; 3 Institute for Clinical Chemistry and Laboratory Medicine, University Hospital Jena, Jena, Germany; 4 Laboratorio de Genética Molecular, Hospital Central Universitario de Asturias, Oviedo, Spain; 5 Estonian Biocentre, Tartu, Estonia; 6 Centre for Neurological Diseases, West Tallinn Central Hospital, Tallinn, Estonia; 7 International Agency for Research on Cancer, Lyon, France; 8 Genomic Epidemiology Group, German Cancer Research Center (DKFZ), Heidelberg, Germany; 9 Estonian Genome Project of University of Tartu, Tartu, Estonia

## Abstract

**Background:**

Hereditary spastic paraplegia (HSP) is a clinically and genetically heterogeneous disorder that can be an autosomal-dominant, autosomal-recessive, or X-linked disease. The most common autosomal-dominant form of the disease derives from mutations in the *SPAST *gene.

**Methods:**

The aim of this study was to analyze 49 patients diagnosed with HSP from the Estonian population for sequence variants of the *SPAST *gene and to describe the associated phenotypes. Healthy control individuals (n = 100) with no family history of HSP were also analyzed. All patient samples were screened using denaturing high performance liquid chromatography (DHPLC) and multiplex ligation-dependent probe amplification (MLPA) assay. Samples with abnormal DHPLC and MLPA profiles were sequenced, with the same regions sequenced in control samples.

**Results:**

Sequence variants of *SPAST *were identified in 19/49 HSP patients (38.8%), twelve among them had pathogenic mutations. Within the latter group there was one sporadic case. Eight patients had pure, and four - complex HSP. The twelve variants were identified: seven pathogenic (c.1174-1G>C, c.1185delA, c.1276C>T, c.1352_1356delGAGAA, c.1378C>A, c.1518_1519insTC, c.1841_1842insA) and five non-pathogenic (c.131C>T, c.484G>A, c.685A>G, c.1245+202delG, c.1245+215G>C). Only 2 of these mutations had previously been described (c.131C>T, c.1245+202delG). Three mutations, c.1174-1G>C, c.1276 C>T, c.1378C>A, showed intrafamilial segregation.

**Conclusion:**

This study identified new variants of the *SPAST *gene which included benign missense variants and short insertions/deletions. No large rearrangements were found. Based on these data, 7 new pathogenic variants of HSP are associated with clinical phenotypes.

## Background

Hereditary spastic paraplegia (HSP) comprises a group of rare neurodegenerative disorders that are characterized by progressive spasticity and weakness in the legs. The prevalence of HSP in European populations has been reported to vary from 0.5 to 12 individuals per 100,000 [1-4]. For Estonia, a relatively small country with a population of 1.3 million, the prevalence of HSP was recently estimated to be 4.4 per 100,000 [5]. Clinical and genetic heterogeneity, as well as variable severity, are characteristic features of HSP [6], a disorder which is further classified as pure (pHSP) or complex (cHSP). In pHSP, spasticity and motor deficit in the legs, brisk reflexes, and Babinski's sign are often accompanied by deep sensory impairment and sphincter disturbances. In contrast, a number of neurological and extra-neurological features are associated with cHSP which include ataxia, distal amyotrophy, optic neuropathy, cognitive impairment, retinopathy, and gastro-oesophageal reflux [7]. Large inter- and intrafamilial variations in the age of symptom onset, disease progression, and extent of disability have also been observed and are typical of this disorder. Furthermore, HSP can be inherited as an autosomal-dominant (AD-HSP), autosomal-recessive (AR-HSP), or rarely, as an X-linked (X-HSP) trait [8]. The number of different loci associated with HSP includes 18 for AD-HSP, 22 for AR-HSP, and 4 for X-linked HSP [7,9-11].

Currently, there are over 150 mutations in the entire *SPAST *gene (also known as *spastin *or *SPG4*) that have been reported to cause at least 40% of all AD-HSP cases [7,12]. In addition, large-scale rearrangements, such as exon deletions, are frequently found to cause HSP, which has been estimated to account for up to 20% of patients with otherwise mutation-negative HSP [13,14]. The spectrum of mutations associated with HSP is compatible with haploinsufficiency being the relevant pathogenic mechanism for this disorder. In addition, there have only been a few benign or unclear missense variants in *SPG4 *and *SPG3A *associated with unknown effects [15,16]. Interestingly, missense mutations have been shown to result in phenotypes that are similar to those of exon rearrangements [7].

The understanding of genotype-phenotype associations for HSP is expanding rapidly, and although mutations in the *SPAST *gene were previously thought to produce only AD-pHSP, recent advances in clinicogenetic research have indicated that the clinical presentation of HSP can be extremely variable as both sporadic cases and cHSP forms have been described [17]. Hence, it is important to expand the available knowledge of HSP. Accordingly, a recent epidemiological study of HSP patients in Estonia provided clinical information for a relatively well-defined population [5]. Therefore, the aim of this study was to detect mutations present in the *SPAST *gene and to characterize the clinical phenotypes of these patients.

## Methods

### Study subjects

This study was approved by the Ethics Review Committee on Human Research of the University of Tartu, Estonia (protocol 110/5, 18.11.2002), and informed consent was obtained from all study participants.

Based on an Estonian epidemiological study that evaluated patients diagnosed with HSP between 2001 and 2005 using a multisource approach [5], and previously described diagnostic criteria for HSP [18,19], 49 patients were included in this study. Twenty-two of the HSP patients belonged to 10 different families, while 10 patients had an unconfirmed family history, and 17 were sporadic cases. All patients were evaluated by at least two experienced neurologists. Neuropsychological evaluations (i.e. Brief Repeatable Battery of Neuropsychological Tests, Beck Depression Inventory, Mini-Mental State Examinations) were performed. Urinary dysfunction was also evaluated and blood samples were collected. Healthy individuals (n = 100) with no family history of HSP those were older than 45 years were used as population controls. All samples were coded. Data for the study participants are presented in Table 1.

**Table 1:** Study participant data.

	Patients (n = 49)	Controls (n = 100)
**Gender**		
Male	32	50
Female	17	50
**Mean age**		
Years (range)	50 (11-75)	64 (45-90)
**Nationality**		
Estonian	39	97
Russian	7	3
Other	3	0

### DNA extraction and analysis of sequence variants

DNA extraction from whole blood was performed using a High Pure PCR Template Preparation Kit (Roche Diagnostics GmbH, Mannheim, Germany). Previously described PCR primers were used for the analysis of the 17 exons and splice sites of the *SPAST *gene [20]. PCR products of all 49 samples were screened using denaturing high performance liquid chromatography (DHPLC), and *SPAST *copy number aberrations were detected using multiplex ligation-dependent probe amplification (MLPA) assays (P165, MRC-Holland, The Netherlands) as previously described [13]. Only sporadic cases with normal DHPLC profiles were not sequenced. The same regions in both HSP and control samples were sequenced using the BigDye^® ^Terminator v3.1 Cycle Sequencing Kit (Applied Biosystems, Foster City, CA, USA). ChromasPro 1.34 http://www.technelysium.com.au/ChromasPro.html was used for sequence analysis.

### Statistical analysis

Differences between patient clinical parameters were detected using a 2-tailed T-test (Microsoft^® ^Office Excel 2003).

## Results

### Genetic analysis of the *SPAST *gene

According to sequencing results, in 19/49 (38.8%) individuals, 12 nucleotide changes were detected, of which 10 were new (Table 2). All of the individuals were heterozygous for the detected sequence variants without gender predisposition. There were five non-pathogenic and seven presumably pathogenic variants (mutations). One new sequence variant, c.1245+215G>C, and a previously described variant, c.1245+202delG, were detected in both HSP patients and controls. Therefore, both of these intronic variants were considered benign single nucleotide polymorphisms (SNPs). Three pathogenic mutations, c.1174-1G>C, c.1276 C>T and c.1378C>A, showed intrafamilial segregation. All other probable pathogenic mutations (i.e. c.1185delA, c.1352_1356delGAGAA, c.1518_1519insTC, and c.1841_1842insA) were detected in index patients.

**Table 2:** Description of *SPAST *gene variants identified in individuals with HSP.

Variant^#^	Identified by	Location	Predicted effect at the protein level^#^	Present in (49 patients/ 100 controls)	Patients	Intrafamilial segregation	Inferred pathogeneity
c.131C>T*	S	exon 1	p.S44L	2/0	2942, 2943	-	NP

c.484G>A	DHPLC/S	exon 2	p.V162I	3/0	2627, 2747, 2943	-	NP

c.685A>G	DHPLC/S	exon 5	p.S229G	1/0	2930	-	NP

c.1174-1G>C	S	intron 8	missplicing (deletion exon 9?)	3/0	2109, 2930, 2931	Yes	P

c.1185delA	DHPLC/S	exon 9	p.V385VfsX11	1/0	2752	-	P

c.1276 C>T	S	exon 10	p.L426F	3/0	2388, 2747, 2754	Yes	P

c.1245+202delG*	S	intron 10	none	3/4	2321, 2386, 2750	-	NP

c.1245+215G>C	S	intron 10	none	1/2	2960	-	NP

c.1352_1356del GAGAA	DHPLC/MLPA/S	exon 11	p.R451RfsX5	1/0	2753	-	P

c.1378C>A	DHPLC/S	exon 11	p.R460S	2/0	2480, 2482	Yes	P

c.1518_1519insTC	MLPA/S	exon 13	p.S507SfsX23	1/0	2478	-	P

c.1841_1842insA	DHPLC/S	exon17	p.T614NfsX no Stop codon	1/0	2389	-	P

^#^nomenclature according to HGVS http://www.hgvs.org/mutnomen/; *previously described; DHPLC - denaturing high performance liquid chromatography; MLPA - multiplex ligation-dependent probe amplification; S - sequencing; P - pathogenic; NP - non-pathogenic.

### Phenotypes of HSP patients with *SPAST *gene mutations

Pathogenic mutations in the *SPAST *gene were detected in 12 individuals diagnosed with HSP (Table 3). Nine patients with AD-HSP belonged to four different pedigrees: patients 2109, 2930 and 2931 to pedigree I, patients 2480 and 2482 to pedigree II, patients 2833, 2747 and 2754 to pedigree III and patient 2389 to pedigree IV. There was one clinically confirmed sporadic case (patient 2478). Two subjects with HSP had an unconfirmed family history (patients 2752 and 2753). Patient 2753 was a Russian male with a brother living abroad that exhibited the same walking pattern yet had not been evaluated by neurologists and therefore had not been diagnosed with HSP. Yet another patient was an Armenian male (patient 2752) with an unconfirmed family history of HSP and potentially affected relatives living abroad.

**Table 3:** Phenotypes of HSP patients with pathogenic *SPAST *mutations.

Patient	Gender	Nationality	Clinical form of HSP	Age of onset (years)	Additional clinical description	Pedigree	Variant
2109	F	Estonian	AD-cHSP	30	Bladder dysfunction, mild dementia, mild depression	I	c.1174-1G>C
2930	F	Estonian	AD-pHSP	35	Bladder dysfunction, mild depression	I	
2931	F	Estonian	AD-pHSP	10	-	I	

2480	F	Estonian	AD-pHSP	28	Bladder dysfunction, *pes cavus*, moderate depression, uses cane	II	c.1378C>A
2482	M	Estonian	AD-pHSP	3	Mild depression	II	

2388	F	Estonian	AD-pHSP	40	Bladder dysfunction, mild depression, uses cane	III	c.1276 C>T
2747	M	Estonian	AD-cHSP	21	Mild cognitive impairment, moderate depression	III	
2754	F	Estonian	AD-pHSP	12	Bladder dysfunction, mild depression, uses bilateral crutches	III	

2389	F	Estonian	AD-cHSP	46	Bladder dysfunction, mild cognitive impairment, mild depression	IV	c.1841_1842insA

2753	M	Russian	pHSP	36	-	NA	c.1352_1356del GAGAA

2752	M	Armenian	cHSP	38	Bladder dysfunction, mild cognitive impairment, mild depression, uses wheelchair	NA	c.1185delA

2478	M	Estonian	pHSP	35	Sporadic case, bladder dysfunction, uses cane	NA	c.1518_1519insTC

F - female; M - male; HSP - hereditary spastic paraplegia; pHSP - pure HSP; cHSP - complex HSP; AD - autosomal dominant; NA - not applicable.

All patients with pathogenic mutations in the *SPAST *gene exhibited progressive spastic paraparesis, with 8 patients, including the sporadic patient case, also experiencing bladder disturbances (66%) and 9 having mild or moderate degree of depression (75%). Furthermore, 8 patients with pathogenic *SPAST *mutations had pHSP and 4 were diagnosed with cHSP and exhibited different degrees of cognitive impairment (33%). There were 3 patients having both - cognitive decline and bladder disturbances (25%) and they were also depressed.

Two females from pedigree III used assistive devices: a 59-year-old patient (2388) used a cane, and a 40-year-old patient (2754) used bilateral crutches. In addition, an Armenian patient (2752) experienced severe neurological effects from cHSP and required a wheelchair, a 70-year-old male (2478) was classified as a sporadic case and used a cane for walking, while a 57-year-old female (patient 2480 from pedigree II) with pHSP had *pes cavus *and used a unilateral cane. The remaining patients (2109, 2389, 2482, 2747, 2753, 2930, and 2931) walked independently.

## Discussion

Mutations in the *SPAST *gene are the most common genetic abnormality associated with HSP. In this study, 12 changes in the *SPAST *gene were identified, 7 of which represented new pathogenic variants and 2 were previously described. Both missense mutations in the exons (amino acid change) and frameshift mutations (formation of new stop codon) were predominantly identified, which have the potential to alter the protein structure of *SPAST*. Changes in splice sites are also important and can lead to exon skipping and a reduced stability for aberrantly spliced mRNAs [21,22]. Interestingly, no single deletion or duplication of an exon was detected. Based on previous estimates [13-15] and considering our identification of seven pathogenic "small" mutations, one could have expected to find several exonic rearrangements. The lack of this kind of mutations is hypothesized to be a unique aspect of the Estonian HSP population. A presence in additional patients, however, cannot be excluded.

There were 5 non-pathogenic variants in our study group. Two out of three members (patients 2942 and 2943) of one family without pathogenic *SPAST *mutations had a substitution c.131C>T. It has previously been suggested that c.131C>T is a benign SNP, yet represents an aggravating disease modifier, since it is usually associated with a pathogenic variant [23,24]. One previously described variant, c.1245+202delG, was identified as a SNP in the HSP patients analyzed as well as in controls [25]. Another sequence variant, c.1245+215G>C, was not previously reported, but since it was detected in both patients and controls, it is also hypothesized to be a SNP. We would also hypothesize that c.484G>A and c.685A>G represent benign missense variants that are rare and specific to the Estonian population.

In our study group, 2 families contained 2 variants in their *SPAST *gene. For a 33-year-old man with AD-cHSP (patient 2747 from pedigree III), his two affected relatives (his sister and mother - patients 2754 and 2388, respectively) did not have the same sequence variants present in exon 2, yet all affected members of this family had a mutation present in exon 10. Also in one 49-year-old woman (patient 2930 from pedigree I) two variants were found - like two of her relatives with HSP (sister and daughter - patients 2109 and 2931 respectively), she had a splicing mutation at the border of intron 8/exon 9 of the *SPAST *gene, but additionally a change in exon 5. Hence, these two families contain two mutations in their *SPAST *gene, one of which is hypothesized to be *de novo *or a rare SNP. The lack of family segregation of the variants in these pedigrees may be indicative of the non-pathogenic effect of the missense mutations detected in exons 1, 2, and 5 in the Estonian population.

The present study describes phenotypes of HSP patients with *SPAST *gene mutations. By comparing patient phenotypes, the average age of symptom onset for Estonian patients with *SPAST *mutations was determined to be 27.8 years (range 3-46), while in other patients with HSP it was 30.0 years (range 5-69) [5]. The mean difference in the age of onset between the two groups was 2.2 years, which was not determined to be significant. Similarly, for previously published data on 356 patients with known mutations in the *SPAST *gene, no correlation between the age of onset and the type of mutation present could be identified [26]. There was no gender predisposition for patients with *SPAST *mutations, which included 5 males and 7 females. Previous reports regarding gender have been inconsistent, although studies of large Brazilian pedigrees have found that males were more severely affected by HSP [27,28]. The patients with mutations in the *SPAST *gene are less likely to have cHSP, and these data further imply that all patients with HSP should be preferentially tested for *SPAST *mutations. Neurologic co-symptoms associated with patients with *SPAST *mutations were mainly bladder disturbances, cognitive impairment and depression, being discussed in detail elsewhere [29,30]. Compared with previous reports of HSP patients with bladder dysfunction, only a few other authors described a similar co-existence for HSP with neuropsychological symptoms [19]. The clinical relevance of these observations is that patients with *SPAST *mutations should receive a more thorough neurological evaluation so that co-symptoms are diagnosed adequately since their symptoms can often be effectively treated.

The limitations of this study should also be considered. For example, samples from all HSP patients identified in the Estonian population studied were unable to be sequenced, which would have increased the confidence of the conclusions of this study. Furthermore, the use of DHPLC to detect changes in the *SPAST *gene did not reliably identify all of the individuals with abnormal profiles. For example the MLPA assay detected two base pair insertions which were not detected by DHPLC. These differences were confirmed by sequencing. Hence, *SPAST *variants, especially among sporadic cases, could be missed if DHPLC is the only detection method used. In addition, although healthy controls without any history of HSP in their pedigrees were included in this study, it is still theoretically possible that these controls could develop symptoms of HSP when they are older, although it is extremely unlikely.

## Conclusions

In conclusion, the present study describes novel mutations in the *SPAST *gene of HSP patients, thereby confirming the genetic variability associated with this disorder. A lack of exon deletions/duplications and the presence of rare coding SNPs differentiate this Estonian study group from others previously reported in the literature. Due to the large clinical and genetic variabilities observed [31], and the absence of strict genotype-phenotype correlations, we suggest that in the clinical setting it is insufficient to test individuals with HSP for only known *SPAST *mutations, and in the case of negative results, additional loci should be sequenced in case other HSP mutations may be present.

## Competing interests

The authors declare that they have no competing interests.

## Authors' contributions

Acquired, systemized and controlled the data: MB, RT, SML, KGP, SH. Conceived and designed the investigations: ChB, ER, AM, FC. Performed the investigations and experiments: MB, RT, ER, ESF, CaB. Analyzed the data: MB, RT, ChB, ER, FC, CaB, AM. Wrote the paper: MB, RT. Supervised: AM, SH. The first two authors (MB and RT) contributed equally to the production of this article. All authors read and approved the final manuscript.

## Pre-publication history

The pre-publication history for this paper can be accessed here:

http://www.biomedcentral.com/1471-2377/10/17/prepub
